# Preference of trees for nest building by critically endangered white‐rumped vultures (*Gyps bengalensis*) in Nepal

**DOI:** 10.1002/ece3.11175

**Published:** 2024-03-18

**Authors:** Ramji Gautam, Nabin Baral, Hari Prasad Sharma

**Affiliations:** ^1^ Central Department of Zoology, Institute of Science and Technology Tribhuvan University Kathmandu Nepal; ^2^ Department of Zoology, Prithvi Narayan Campus Tribhuvan University Pokhara Nepal; ^3^ School of Environmental and Forest Sciences University of Washington Seattle Washington USA

**Keywords:** bird, breeding success, habitat conservation, nesting tree, nests

## Abstract

White‐rumped vultures (*Gyps bengalensis*) are critically endangered species, and protecting their habitats, particularly the nesting trees, may have a positive impact on their reproductive success. For a better understanding of vultures' habitat needs, the characteristics of nesting trees should be accounted. In this paper, we compare the characteristics of the trees that have vultures' nests and that do not by randomly select a control tree within a 10 m radius of the nesting tree. We extensively searched and monitored the white‐rumped vultures' nests, nesting trees, and nesting tree species in Nepal between 2002 and 2022, and measured the characteristics of sampled trees such as their height, girth, canopy spread, branching orders, and whorls. We recorded 1161 nests of white‐rumped vulture in total on 194 trees belonging to 19 species over the past two decades. White‐rumped vultures preferred the kapok trees (*Bombax ceiba*) for nest construction than other tree species (*χ* ^2^ = 115.38, df = 1, *p* < .001) as 66.49% of nests were built on them. In the logistic regression model, the number of whorls on a tree, canopy spread, and the height of the first branch determined whether a nest was present or absent on a tree. These results help to prioritize the tree attributes in a habitat conservation plan for vultures.

## Graphical abstract

White‐rumped vultures preferred the kapok tree (*Bombax ceiba*) for nest construction over other tree species. White‐rumped vultures preferred those trees which were taller in height, broader in girth wider in canopy spread, had more branching order, and tree whorl for nesting.

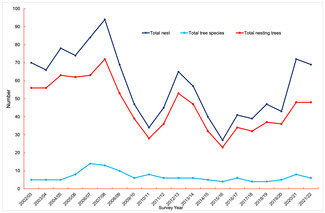

## 1 INTRODUCTION

The characterization of nesting habitats plays a crucial role in comprehending the habitat needs of vultures (Mölder et al., 2020; Yarrow, 2009). An in‐depth knowledge of the characteristics of preferred nesting trees and nest survival is key factor in determining the reproductive success of vultures (Chiavacci et al., 2014; Francis et al., 2011). Predation, natural disasters such as storms, and anthropogenic disturbances such as cutting down the nesting trees and collection of chicks are some reasons for nesting failure in vultures (Baral et al., 2005; Baral & Gautam, 2007; Keyser, 2002; Newton, 1998). The availability of large nesting and roosting trees is crucial for vulture survival as they provide a good vantage point to locate food sources and reduce the risk of predation by land animals (Kendall et al., 2018; Vogel et al., 2014).

The logging of large trees and habitat loss are major threats to the survival of vulture species as they lead to reproductive failure (Fletcher et al., 2018; He & Hubbell, 2011). In Nepal, three out of nine vulture species, namely red‐headed vulture (*Sarcogyps calvus*), slender‐billed vulture (*Gyps tenuirostris*), and white‐rumped vulture (*G. bengalensis*) are typically tree nesters while the Egyptian vulture (*Neophron percnopterus*) builds nests on trees on rare occasions (Ali & Ripley, 1987; Chhangani, 2002). These species require mature and tall trees for nesting (Ahmad et al., 2020; Ghimire et al., 2019; Majgaonkar et al., 2018; Siders & Kennedy, 1996; Thakur, 2015). The wild vulture population has been declining in South Asia due to the use of diclofenac in veterinary practices (Ahmad et al., 2020; Chaudhary et al., 2012; Khan, 2013; Prakash et al., 2007), food shortage (Shah et al., 2019), unintentional poisoning (Clements et al., 2013), human persecution (Clements et al., 2013; Hla et al., 2011), collision with power lines and electrocution (Hamal et al., 2023), and breeding habitat loss (Gautam & Baral, 2013; Hla et al., 2011). As a result, the Nepalese government banned the production, distribution, and sale of diclofenac for veterinary use in 2006 (Prakash et al., 2012). To support the recovery of vulture populations, various conservation programs have been initiated, including captive breeding and release programs, the establishment of vulture safe feeding sites and zones, public awareness campaigns, and the declaration of diclofenac‐free zones (DNPWC, 2015). Making sure the availability of suitable nesting and roosting trees in the wild is crucial for the long‐term survival of vultures (Baral et al., 2013; Pain et al., 2003). However, insufficient information exists regarding what constitutes suitable trees for nesting or roosting. The problem is further exacerbated when the existing nesting trees are cut down to implement development activities such as road and building construction in Nepal (Gautam & Baral, 2013).

In Nepal, white‐rumped vultures are found below 1500 m above sea level, and they were the most abundant and widespread species prior to the 1990s (Ali & Ripley, 1987; Grimmett et al., 2016). White‐rumped vulture has been listed as a critically endangered species in the Red Data Book by BirdLife International due to the drastic population decrease in the wild since 2000 (BirdLife International, 2021). Following the significant drop in wild population, the white‐rumped vulture is now found patchily distributed across Nepal (Baral et al., 2005; Bhusal et al., 2020; Dhakal et al., 2022; Gautam & Baral, 2013; Rana et al., 2019). Despite the effective ban on diclofenac, the species continues to suffer from food shortage, human persecution, unintended poisoning, and habitat destruction (Baral et al., 2005; BirdLife International, 2021), with the latter being the primary cause of local colony extinction due to habitat loss (Baral et al., 2005, 2013). The choice of tree species for nesting by white‐rumped vultures can vary between sites (Bhusal et al., 2023; Ghimire et al., 2019). They usually prefer to nest on tall and mature trees (Bhusal et al., 2020; Ghimire et al., 2019; Subedi, 2008). Providing information on the characteristics of preferred trees sheds light on the ecological requirements of vultures (Sharma et al., 2023) and helps in formulating effective vulture conservation strategies (Beyer & Manica, 2020; Majgaonkar et al., 2018). Furthermore, managers, policymakers, and scholars can have a better understanding of the specific habitat needs of vultures (Polak, 2016) and determine the most suitable habitats for conservation efforts. Therefore, our objective is to undertake a comprehensive evaluation of the characteristics of nesting trees with the goal of informing future habitat restoration and management initiatives for fostering the rejuvenation of the declining population of white‐rumped vultures in the wild.

## 2 METHODS

### 2.1 Study area

**FIGURE 1:**
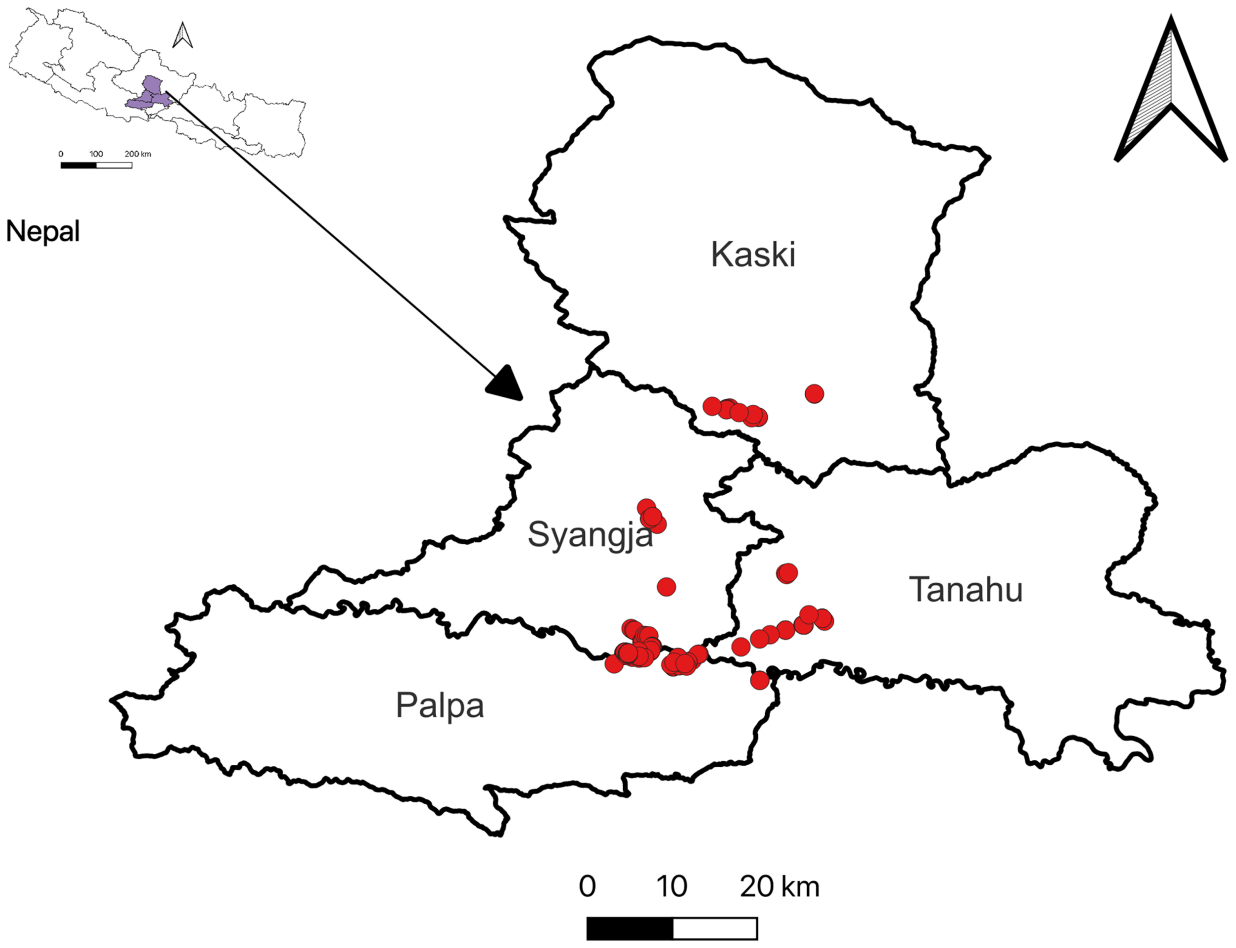
Location of white‐rumped vultures' colonies within four districts (dots indicate study area) in Nepal.

We conducted the study in the west Nepal's Kaski, Syangja, Tanahu, and Palpa Districts (83°15′ to 84°46′ E and 27°26′ to 28°36′ N) that comprised a total area of 5996 km^2^ (Figure 1). The study area has a tropical to sub‐tropical monsoonal climate, with the rainy season extending from June to September, cool‐dry winter from October to January, and hot‐dry summer from February to May. The absolute maximum and minimum temperature of the study area was 40.2 and 0.5°C recorded in May 2007 and January 2013, respectively. In the same period (2001–2020), the total amount of annual precipitation ranged from 2142.4 mm in 2005 to 3871.58 mm in 2020 and the average annual precipitation was 2661.22 mm. The elevation of white‐rumped vulture nesting colonies ranged between 303 and 1168 m above sea level. The study area supports a mixed forest of sal (*Shorea robusta*), chestnut (*Castanopsis indica*), and needle wood (*Schima wallichii*). The majority of nesting trees used by white‐rumped vultures were within the community forests area.

### 2.2 Data collection

Between 2002 and 2022, we recorded the number of nests of white‐rumped vultures in the study area following the protocols used by Baral et al. (2005, 2013) and Gautam and Baral (2013). We marked the location of nests, and noted the nesting tree species. In 2022, we recorded the tree characteristics such as the girth at breast height, longest branching order, number of tree whorls, canopy spread, tree height, first branch height, nest height, and nest branching order for those trees where white‐rumped vulture constructed the nest. We identified tree species in the field and herbarium were made for unidentified tree species which were later identified by the experts at the Central Department of Botany, Tribhuvan University, Kirtipur, Nepal.

In this study, we established a 10 m radius plot around the nesting tree, and randomly selected non‐nesting trees based on preset 160 random angles (0–360° in the increment of 5°) (Yang & Burkhart, 2019). If the non‐nesting tree was absent at the preset random angle, we used the next random angle to locate a tree. If a tree was absent in the second iteration, we did not measure the non‐nesting trees. We recorded the spatial coordinates of the nesting and non‐resting trees using a hand‐held Geographical Position System (GPS; accuracy <5 m). In this way, a randomly selected control pair was established for each nesting tree to compare and contrast their characteristics.

In addition, we also tallied the number of nesting trees lost in the vulture colonies between 2002 and 2022. We also attempted to determine the causes of nesting tree loss, and categorized them into natural and anthropogenic causes.

### 2.3 Variable measurement

In our study, we measured several characteristics of the trees, including the girth at breast height (GBH), longest branching order (LBO), tree whorl (TW), canopy spread (CS), tree height (TH), and the first branch height (FBH). For white‐rumped vulture nesting trees, we additionally measured the nest branching order (NBW), nesting whorl (NW), and nest height (NH) (See Table S1).

#### 2.3.1 Girth at breast height

It is the measurement of the circumference of the tree trunk at a standard height of 1.5 m from the ground surface, which was accurately determined using a measuring tape.

#### 2.3.2 Longest branching order

The branching order is the arrangement of the tree branches or segments. Among the various methods applied for measuring the longest branching order, we used a centrifugal system instead of numbering the branching order from the tip toward the stem of the tree segments (Horton, 1945; Strahler, 1957; Uylings et al., 1975). We recorded Order 1 axis (the trunk), Order 2 axis (the branch growing directly from the trunk), Order 3 axis (branches growing on Order 2 axes), and so on. We used the centrifugal ordering system to locate nests and the longest branching pattern of the trees. We excluded the dead, broken, pruned, or logged branches for this recording.

#### 2.3.3 Tree whorls

We recorded the total number of whorls by counting the presence of live branches arising from the main tree axis or main stem (Kidombo & Dean, 2018). We excluded the node without live branches and the dead main axis. However, we recorded the whorl as a one whorl tree if no live branches are present below and above the branches. We recorded the first branch as the first whorl, successively upward second, third, and so on.

#### 2.3.4 Canopy spread

We measured the longest and shortest canopy spread of the tree using a measuring tape. We measured the widest crown spread from the ground at the longest axis of the crown and the shortest crown spread by making a right angle to the widest crown spread following Blozan (2006). The average of the widest and shortest extents of the crown was used for the canopy spread.

#### 2.3.5 Tree height

The tree height is the measure of a tree from the ground to the top. We measured the tree height with the help of a clinometer and a measuring tape. We measured the distance from the researcher to the tree. The clinometer was used to observe the treetop, and the angle of inclination was noted. A 1.5 m high stick was used to take angle with the help of a clinometer. We used the formula, i.e. height of the tree (m) = (basal distance in meter × tan*α*) + 1.5 m (where, *α* is the angle of the treetop).

#### 2.3.6 First branch height

The first branch height is the measure of height from the ground to the first live branch. We applied the same procedure for the tree height (sans direct measurement).

#### 2.3.7 Nest branching order

Like the branching order, we used the centrifugal method to find the nesting branching order if the branch has a nest. We recorded nesting branching Order 1, if the nest was located on the trunk. Further, if the nest was located on the branch raised from the trunk, it was noted as two and so on (Suzuki & Suzuki, 2009).

#### 2.3.8 Nest whorl

The nest whorl was counted from lower to upper region of a tree. If the nest was present on the branch developed from the first whorl, it was considered as the nest present on the first whorl. If the nesting branch was from the second whorl, it was considered as a second nesting whorl and so on.

#### 2.3.9 Nest height

We measured the nest height as a distance from the ground to the nest. We measured the nest height with the help of a clinometer and a measuring tape as applied in the measurement of tree height.

### 2.4 Data analysis

We tested the data for normality test using the Shapiro–Wilk test and most of the variables were not normally distributed. Therefore, we performed the Mann–Whitney *U* test to examine whether the characteristics of nesting and non‐nesting trees differ (Neuhäuser, 2010). After performing bivariate analyses, we built a logistic regression model to determine the relative strength of the variables that influence whether a nest is built on a tree or not. The data met the major assumptions required for logistic regression. Taking the binary presence or absence of nest on a tree as a response variable, and the explanatory variables such as the girth at breast height, longest branching order, tree whorl, canopy spread, tree height, and the first branch height, we fitted the logistic regression model. After fitting the model, we examined the variance inflation factor for multicollinearity, which was found to be <10 (O'brien, 2007; Schreiber‐Gregory et al., 2018) (Table S2). We performed the DHARMa Moran's *I* test for distance‐based autocorrelation (Hartig, 2022), and found no statistically significant autocorrelation in the spatial distribution. All analyses were performed in the R Program (R Core Team, 2022).

## 3 RESULTS

**FIGURE 2:**
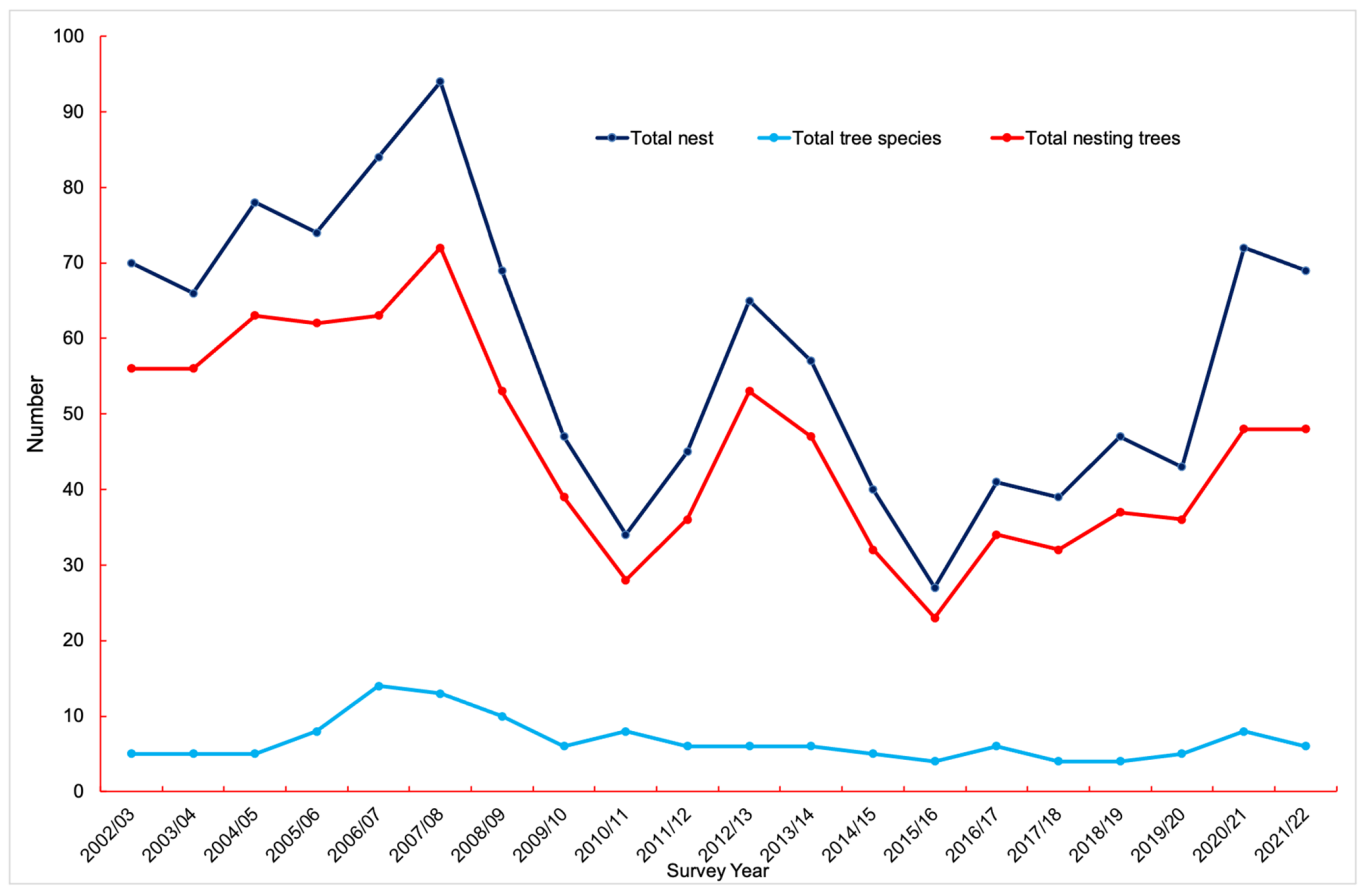
Number of nesting trees, tree species, and nests of white‐rumped vulture in Nepal during the breeding season between 2002/03 and 2021/22.

We recorded a total of 1161 white‐rumped vulture nests on 194 individual trees belonging to 19 distinct tree species in the study area between 2002 and 2022 (Tables S3 and S4). The number of nests and the total number of nesting trees showed a *U*‐shaped trend over the study period, meaning that their number declined in the middle range of the study period. The total number of tree species used for nesting had remained more or less constant throughout the study period (Figure 2).

The kapok tree (*Bombax ceiba*) harbored the highest number of nests (66.49%, *n* = 129) followed by quassia wood trees (*Picrasma javanica*) (7.22%, *n* = 14), mango trees (*Magnifera indica*) (3.61%, *n* = 7), silver gray wood (*Terminalia tomentosa*) (3.09%, *n* = 6), khair (*Acacia catechu*) (2.58%, *n* = 5), and the other 14 different tree species (12.88%, *n* = 25). Kapok trees were more preferred for nest construction by white‐rumped vulture as compared to other tree species (*χ* ^2^ = 115.38, df = 1, *p* < .001). Among the 45 tree species recorded in the white‐rumped vulture colonies, comparatively fewer tree species (42.22%, *n* = 19) were utilized by the vultures for nesting, while the remaining tree species (57.78%, *n* = 26) were not utilized for nesting (Table S5).

Of the 194 nesting trees, most trees (66.49%, *n* = 129) had one nest followed by trees with two nests (26.80%, *n* = 52), three nests (4.12%, *n* = 8), four nests (1.03%, *n* = 2), and five nests (1.55%, *n* = 3).

### 3.1 Tree characteristics between nesting and non‐nesting trees

Among 194 nesting trees, we measured the characteristics of 112 nesting trees because 48 trees were lost and 34 trees were difficult to access due to topographical conditions. In the case of non‐nesting trees, we measured only 86 trees because, in the remaining 26 cases, there were not any trees for the measurement. The characteristics of white‐rumped vulture nesting and non‐nesting trees were found to vary (Table 1). The average girth at breast height (GBH) of trees in the study area was 2.57 ± 1.93 m (nesting tree: 3.95 ± 1.43 m; non‐nesting tree: 0.76 ± 0.33 m). The average highest number of branching pattern order of trees was 9.70 ± 3.04 (nesting tree: 10.91 ± 2.58; non‐nesting tree: 8.10 ± 2.87). The average number of tree whorl was found to be 6.23 ± 3.38 (nesting tree: 7.31 ± 2.86; non‐nesting tree: 4.82 ± 3.49), and the average tree canopy spread 13.56 ± was 7.83 m (nesting tree: 19.08 ± 5.72 m; non‐nesting tree: 6.38 ± 2.65 m). The average height of trees in the study area was 18.43 ± 7.40 m (nesting tree: 23.61 ± 5.79 m; non‐nesting tree: 12.03 ± 4.87 m) and the average first branch height was 6.93 ± 4.70 m (nesting tree: 9.71 ± 4.01 m; non‐nesting tree: 3.33 ± 2.60 m).

**TABLE 1:** Comparison between white‐rumped vulture nesting and non‐resting trees in Nepal.

Variable	Nesting tree (*n* = 112)		Non‐nesting tree (*n* = 86)		Mann–Whitney *U* test	*p*
	Mean (SD)	Range	Mean (SD)	Range		
Girth at breast height	3.95 (1.43)	1.26–8.50	0.76 (0.33)	0.30–2.10	9615.5	.001
Longest branching order	10.91 (2.58)	6–19	8.10 (2.87)	3–14	7217	<.001
Tree whorl	7.31 (2.86)	1–14	4.82 (3.49)	1–22	7123.5	<.001
Canopy spread	19.08 (5.72)	4.08–37.75	6.38 (2.65)	1.38–13.75	9436.5	<.001
Tree height	23.61 (5.79)	13.42–36.37	12.03 (4.87)	3.00–25.06	9134	<.001
First branch height	9.71 (4.01)	0.80–18.30	3.33 (2.60)	0.40–11.50	8608.5	<.001
Nest branching order	2.91 (1.83)	1–13	–	–	–	–
Nesting whorl	5.68 (0.27)	1–13	–	–	–	–
Nest height	18.46 (0.42)	8.50–36.12	–	–	–	–

*Note*: Variables girth at breast height (m), longest branching order (n), tree whorl (number), canopy spread (m) tree height (m), first branch height (m), nest branching order (number), nest branching whorl (number), and nest height (m). “–” Non‐nesting trees don't have nests.

The number of tree whorls, canopy spread, and the first branch height were statistically significant explanatory variables to determine whether a nest would be built on a tree. The odds of a tree harboring a nest increased with the number of tree whorls and the increasing height of the first branch, but the odds decreased with the wider canopy spread (Table 2).

**TABLE 2:** Logistic linear regression model for factors affecting the occurrence of nest of white‐rumped vulture in Nepal.

Variable	Estimate	SE	*t*	*p*
Intercept	−0.328	0.075	−4.373	.001
Girth at breast height	0.003	0.004	0.727	.468
Longest branching order	0.005	0.008	0.581	.562
Tree whorl	0.021	0.006	3.404	.001
Tree height	0.002	0.005	0.47	.638
First branch height	0.035	0.005	7.796	<.001
Canopy spread	−0.328	0.075	−4.373	.001

*Note*: The response variable is the presence or absence of nests on a tree.

### 3.2 Available nesting trees around 10‐m radius of the nesting trees

Within the 10 m radius of the nesting trees, we recorded 969 trees belonging to 45 species (Table S5). The most frequent tree was rohituka (*Aphanamixis polystachya*) (15.48%, *n* = 150) which was followed by small flower crape myrtle (*Lagerstromia parviflora*) (12.07%, *n* = 117), tiger's milk spruce (*Sapium insigne*) (10.63%, *n* = 103), potka siris (*Albizia lucidior*) (8.98%, *n* = 87), fever pod (*Holarrhena pubescens*) (5.88%, *n* = 57), bhellar (*Trewia nudiflora*) (5.78%, *n* = 56), karma (*Adina cordifolia*) (4.95%, *n* = 48), garuga (*Garuga pinnata*) (4.44%, *n* = 43), sage‐leaved alangium (*Alangium salviifolium*) (3.30%, *n* = 32), and wind killer (*Premna integrifolia*) (2.79%, *n* = 27). There were other less frequent 249 trees belonging to 35 species.

### 3.3 Nesting tree lost

The white‐rumped vultures used more trees for nesting in community forests (60.82%) than in the vicinity of human settlements (39.17%) during the study period. Of the 194 nesting trees, 48 nesting trees were lost during the study period: 24 nesting trees were cut down in community forests and 24 nesting trees in the vicinity of human settlements. Humans cut down 33 nesting trees while 15 nesting trees were destroyed by natural causes such as old age and storms. Among the lost tree species, most (20 trees) belonged to kapok followed by quassia wood (8), mango (6), khiar (3), red cedar (3), garuga (2), sacred Fig (2), sal (2), baheda (*Terminalia bellerica*) (1), and butter tree (1).

## 4 DISCUSSION

White‐rumped vultures are a selective tree nesting species, and their choice of nesting trees may be influenced by safety considerations. In the study area, rohituka trees are the most abundant trees, but vultures used kapok trees for building nests most often. Previous studies conducted document that white‐rumped vultures tend to use crocodile bark tree (*Terminalia tomentosa*) and sal for nest building in lowland Nepal (Bhusal et al., 2020; Subedi, 2008), but chir pine (*Pinus roxburghii*) and kapok are found to be the most commonly occurring nesting trees for this species in mid hills (Rana et al., 2019). In India, white‐rumped vultures use chir pine (*Pinus roxburghii*) for nesting in Himanchal Pradesh (Thakur, 2015), coconut palm (*Cocos nucifera*), mango (*Magnifera species*) and *Termenalia* in Western Maharashtra (Majgaonkar et al., 2018), and white murdah (*T. arjua*) in Tamilnadu (Ramakrishnan et al., 2014). It appears that white‐rumped vultures preferred certain trees irrespective of the diversity and availability of other trees. In Nepal, the availability of suitable nesting and roosting habitats may be a limiting factor, as vulture colonies are often located outside the protected areas.

White‐rumped vultures choose large‐sized trees, and the trees that have the longest branching orders for nest construction. These trees tend to be strong enough to support the vultures' body weight during takeoff and landing. The white‐rumped vultures prefer taller trees for nest construction, so that they can easily detect carcasses directly or observe a long chain of descending vultures toward the carcass (Jackson et al., 2008; Rouviere & Ruxton, 2022). This minimizes the energy expenditure required for scavenging, which can be a crucial factor in maintaining optimal breeding conditions. Overall, it seems that choosing large and mature trees is the best option for nest building and breeding of white‐rumped vultures.

The study suggests that the tree whorl, canopy spread, and first branch height are important predictive variables for white‐rumped vulture nest construction. More tree whorls might provide a wider space for nest placement and roosting to the vultures. That might increase breeding success. Not only white‐rumped vultures, but also black‐crowned night herons (*Nycticorax nycticorax*) construct their nest in those trees that having nine or more whorls (Wood & Wood, 1933). Furthermore, white‐rumped vultures build more nests on trees with low canopy spread, probably that may provide easy access for nest establishment with minimal effort, thereby increasing nesting success and ultimately leading to a higher fledgling success rate (Barash, 1975). The advantage of nesting and roosting on the first branch of the trees extends beyond safety and ease of nest construction.

The white‐rumped vultures are usually found roosting and nesting near human settlements and community forests due to the availability of food, which was supported by livestock farming and open carcasses disposal systems. However, for more economic benefits and infrastructure development, large and mature trees are lost in the study area, which reduces nesting and roosting habitats for vultures (Baral et al., 2005; Gautam & Baral, 2013). To protect white‐rumped vultures, it is necessary to implement measures to conserve nesting and roosting trees, including controlling the harvesting of large and tall trees that have shorter canopy spread and more whorls.

Over the past decade, the total number of white‐rumped vultures' nests has increased in Nepal. It was probably due to diclofenac ban and the establishment of vulture safe feeding sites and zones after 2006 (Prakash et al., 2012). The number of nests in active colonies has a positive effect on the overall nests of the species in Nepal even though some trees which white‐rumped vultures use for nesting are lost in the study area. The white‐rumped vultures exhibit a consistent pattern of reusing nesting trees year after year *(*Ali & Ripley, 1987; Gautam & Baral, 2013). However, we measured the characteristics of both nesting and non‐nesting trees only one time between 2002/03 and 2021/22. This limitation might create some possibility of bias in the relationship between nest construction and the influence of tree characteristics. The reviewers raised an issue of spatial autocorrelation in the attributes of nesting and non‐nesting trees. Trees in the neighborhood might share some similarities, but our focus was on examining why certain trees were chosen to build nests than others, given how similar or different the attributes of the trees found in the neighborhood. Based on the research design, the spatial autocorrelation is statistically controlled in our case.

## AUTHOR CONTRIBUTIONS

**Ramji Gautam:** Conceptualization (equal); data curation (equal); formal analysis (equal); funding acquisition (lead); writing – original draft (equal); writing – review and editing (equal). **Nabin Baral:** Conceptualization (equal); data curation (equal); methodology (equal); supervision (equal); writing – review and editing (equal). **Hari Prasad Sharma:** Conceptualization (equal); data curation (equal); methodology (equal); supervision (equal); writing – review and editing (equal).

## CONFLICT OF INTEREST STATEMENT

Authors declare no conflict of interest.

## Supporting information


Tables S1–S5

## Data Availability

The data are available at Dryad https://doi.org/10.5061/dryad.2ngf1vhtv and https://datadryad.org/stash/share/OhsNL3DyHD53Z3cIspG3J3cSOxv6A9b9EONUlBrVYWs.
