# Supplementary material for: Preference of trees for nest building by critically endangered white‐rumped vultures (Gyps bengalensis) in Nepal
Source: Ecol Evol. 2024 Mar 18;14(3):e11175. doi: 10.1002/ece3.11175 (PMC10948368; doi:10.1002/ece3.11175)
Supplement: Supplementary file 1 — Tables S1–S5 [file ECE3-14-e11175-s001.docx]

Supplementary Table 1. Nesting trees characteristics of the White-rumped Vulture in the study area.

| Variable | Description |
| --- | --- |
| Girth at breast height (GBH) | Measurement of the girth of the tree at breast height (m) |
| Longest branching order (BO) | Measurement of the longest branching pattern of a particular tree |
| Nest branching order (NBO) | The branching pattern order which holds the nest |
| Total number of tree whorl (TW) | Measurement of the total whorl number of a particular tree |
| Canopy spread (CS) | Mean of longest and shortest canopy spread measure of a particular tree(m) |
| Tree height(TH) | The estimated height of a particular tree from the ground (m) |
| First branching height (FBH) | The estimated height of the first branch of a particular tree from the ground (m) |
| Nest height (NH) | The estimated height of the nest from the ground (m) |
| Nest whorl (NH) | Measurement of nest bearing whorl |

Supplementary Table 2. Variance inflation factor between the variables of nesting and available trees around 10 m radius of White-rumped Vulture’s nesting trees in Nepal.

| Variable | VIF |
| --- | --- |
| Girth at Breast Height | 1.11 |
| Longest branching order | 1.74 |
| Tree whorl | 1.04 |
| Canopy spread | 2.64 |
| Tree height | 2.25 |
| First branch height | 1.82 |

Supplementary Table 3. Number of nest per tree used by White-rumped Vulture in Nepal during the breeding season 2002/03-2020/22.

| Survey year | Single nesting tree | Two nesting tree | Thee nesting tree | Four nesting tree | Five nesting tree | Total nesting trees | Total occupied nest |
| --- | --- | --- | --- | --- | --- | --- | --- |
| 2002/03 | 43 | 12 | 1 | 0 | 0 | 56 | 70 |
| 2003/04 | 48 | 6 | 2 | 0 | 0 | 56 | 66 |
| 2004/05 | 52 | 8 | 2 | 1 | 0 | 63 | 78 |
| 2005/06 | 53 | 6 | 3 | 0 | 0 | 62 | 74 |
| 2006/07 | 46 | 15 | 0 | 2 | 0 | 63 | 84 |
| 2007/08 | 57 | 10 | 3 | 2 | 0 | 72 | 94 |
| 2008/09 | 43 | 8 | 1 | 0 | 1 | 53 | 67 |
| 2009/10 | 32 | 6 | 1 | 0 | 0 | 39 | 47 |
| 2010/11 | 23 | 4 | 1 | 0 | 0 | 28 | 34 |
| 2011/12 | 28 | 7 | 1 | 0 | 0 | 36 | 45 |
| 2012/13 | 42 | 9 | 1 | 1 | 0 | 53 | 67 |
| 2013/14 | 38 | 8 | 1 | 0 | 0 | 47 | 57 |
| 2014/15 | 25 | 6 | 1 | 0 | 0 | 32 | 40 |
| 2015/16 | 20 | 2 | 1 | 0 | 0 | 23 | 27 |
| 2016/17 | 29 | 4 | 0 | 1 | 0 | 34 | 41 |
| 2017/18 | 27 | 4 | 0 | 1 | 0 | 32 | 39 |
| 2018/19 | 28 | 8 | 1 | 0 | 0 | 37 | 47 |
| 2019/20 | 30 | 5 | 1 | 0 | 0 | 36 | 43 |
| 2020/21 | 35 | 7 | 3 | 1 | 2 | 48 | 72 |
| 2021/22 | 34 | 10 | 2 | 1 | 1 | 48 | 69 |
| Total | 733 | 145 | 26 | 10 | 4 | 918 | 1161 |

Supplementary Table 4. Nesting tree species of Whit-rumped Vulture and their frequency in west Nepal.

| SN | Local name | Common name | Scientific name | Number |
| --- | --- | --- | --- | --- |
| 1 | Aap | Mango | *Magnifera indica* | 7 |
| 2 | Barro | Baheda | *Terminalia bellerica* | 1 |
| 3 | Chhatiwan | White Cheese Wood | *Alstonia Scholaris* | 3 |
| 4 | Chilaune | Needlewood Tree | *Schima wallichii* | 3 |
| 5 | Chiuri | Butter Tree | *Aesendra butyraceae* | 1 |
| 6 | Dabdabe | Grey Downy Balsam | *Garuga pinnata* | 3 |
| 7 | Karang | Yello Teak | *Adina cardifolia* | 2 |
| 8 | Katush | Chestnut | *Castanopsis indica* | 2 |
| 9 | Kavro | Java Fig | *Ficus lacor* | 3 |
| 10 | Khair | Cutch Tree | *Acacia catechu* | 5 |
| 11 | Lokate | Jungle Cork | *Holoptelia integrifolia* | 1 |
| 12 | Padke | Potka Siris | *Albizia lucidor* | 2 |
| 13 | Peeple | Sacred Fig | *Ficis religiosa* | 3 |
| 14 | Sal | Sal | *Shorea robusta* | 4 |
| 15 | Saj | Silver Grey Wood | *Terminalia tomentosa* | 6 |
| 16 | Simal | Kapok | *Bombax ceiba* | 129 |
| 17 | Swami | Weeping Fig | *Ficus benjamina* | 1 |
| 18 | Tiju | Quassia Wood | *Picrasma javanica* | 14 |
| 19 | Tuni | Redcedar | *Toona ciliata* | 4 |

Supplementary Table 5. Non-nesting tree species found around the nesting trees of White-rumped Vulture in west Nepal’s Kaski, Syangja, Tanahu, and Palpa Districts of Gandaki and Lumbini Province.

| SN | Local name | Common name | Scientific name | Number |
| --- | --- | --- | --- | --- |
| 1 | Chuletro | Chuletro | *Brassaiopsis hainla* | 7 |
| 2 | Chilaune | Needlewood tree | *Schima wallichii* | 26 |
| 3 | Katush | Chest Nut | *Castanopsis indica* | 16 |
| 4 | Kutmiro | Yati | *Listea monopetala* | 16 |
| 5 | Bhalupayile | Chinese Alangium | *Alangium chinense* | 18 |
| 6 | Khirro | Tiger's milk spruce | *Sapium insigne* | 103 |
| 7 | Kurau | Fever pod | *Holarrhena pubescens* | 57 |
| 8 | Bedulo | Bedulo | *Ficus subincisa* | 1 |
| 9 | Tiju | Quassia wood | *Picrasma javanica* | 17 |
| 10 | Aurelu | Aurelu | *Flacourtia* sp. | 9 |
| 11 | Sal | Sal tree | *Shorea robusta* | 8 |
| 12 | Gideri | Wind Killer | *Premna integrifolia* | 27 |
| 13 | Khaniyo | Dropping Fig | *Ficus semicordata* | 5 |
| 14 | Thotne | Hairy Fig | *Ficus hispida* | 15 |
| 15 | Dabdabe | Garuga | *Garuga pinnata* | 43 |
| 16 | Amara | Tree of heaven | *Ailanthus excelasa* | 2 |
| 17 | Saj | Baheda | *Terminalia elliptica* | 24 |
| 18 | Kavro | Java Fig | *Ficus lacor* | 19 |
| 19 | Bar | Banyan | *Ficus benghalensis* | 3 |
| 20 | Rukhakatahar | Jack tree | *Atrocarpus heterophyllus* | 2 |
| 21 | Simal | Kapok | *Bombax ceiba* | 14 |
| 22 | Padke | Potka Siris | *Albizia lucidior* | 87 |
| 23 | Khayar | Cutch | *Acacia catechu* | 10 |
| 24 | Chhatiwan | Black board tree | *Alstonia scholaris* | 3 |
| 25 | Rohini | Red Berry | *Mallotus philippensis* | 4 |
| 26 | Aap | Mango | *Magnifera indica* | 1 |
| 27 | Pipal | Sacred Fig | *Ficus religiosa* | 5 |
| 28 | Lokte/Daje | Jungle cork | *Holoptelia integrifolia* | 6 |
| 29 | Swami | Golden fig | *Ficus benjamina* | 3 |
| 30 | Sunkauli | Indian bay leaf | *Cinnamomum tamala* | 1 |
| 31 | Lakuri | Himalayan Mannaash | *Fraxinus floribundax* | 1 |
| 32 | Badahar | Monkey fruit | *Artocarpus lakoocha* | 2 |
| 33 | Ramriththo | Bhellar | *Trewia nudiflora* | 56 |
| 34 | Lasune | Rohituka tree | *Aphanamixis polystachya* | 150 |
| 35 | Taki | Butterfly tree | *Bauhinia purpurea* | 1 |
| 36 | Budhodhayero | Small flower crape myrtle | *Lagerstromia parviflora* | 117 |
| 37 | Nibuwa | Lemon | *Citrus lemon* | 1 |
| 38 | Pakhuri | Pakhuri | *Ficus glaberrima* | 1 |
| 39 | Tuni | Red cedar | *Cedrela tuna* | 2 |
| 40 | Asare | Sage leaf Alangium | *Alangium salviifolium* | 32 |
| 41 | Karang | Karma | *Adina cordifolia* | 48 |
| 42 | Belapatra | Golden apple | *Aegle marmelos* | 1 |
| 43 | Nibaro | Nibaro | *Ficus rosenbergii* | 2 |
| 44 | Gaya | Gaya | *Bridelia retusa* | 2 |
| 45 | Chiuri | Butter tree | *Aesendra butyraceae* | 1 |
